# Chemometric Analysis of Extracts and Fractions from Green, Oxidized, and Microbial Fermented Teas and Their Correlation to Potential Antioxidant and Anticancer Effects

**DOI:** 10.3390/antiox9101015

**Published:** 2020-10-19

**Authors:** Chan-Su Rha, Young Sung Jung, Jung-Dae Lee, Davin Jang, Mi-Seon Kim, Min-Seuk Lee, Yong Deok Hong, Dae-Ok Kim

**Affiliations:** 1 AMOREPACIFIC R&D Center, Yongin 17074, Korea; hydhong@amorepacific.com; 2 Department of Food Science and Biotechnology, Kyung Hee University, Yongin 17104, Korea; chembio@khu.ac.kr (Y.S.J.); davin1031@khu.ac.kr (D.J.); miseonkim95@khu.ac.kr (M.-S.K.); 3 Osulloc Tea R&D Center, Osulloc Farm Corporation, Seogwipo 63521, Korea; jedlee@osullocfarm.com (J.-D.L.); leems@osullocfarm.com (M.-S.L.)

**Keywords:** anticancer effect, antioxidant capacity, high-performance liquid chromatography-mass spectrometry, fermented green tea, multivariate analysis, oxidized green tea, partial least squares-discriminant analysis

## Abstract

Previous reports on phytochemicals in green tea (GT) and processed teas mainly focused on more representative compounds such as catechins. Here, we focus on the insignificantly studied non-catechin components in tea extracts, and explore the multivariate correlation between diverse phenolic compounds in tea and the in vitro antioxidant and anticancer effects. Extracts from GT and four types of processed teas were further divided into hydrophilic and hydrophobic fractions, whose phenolic compositions and antioxidant capacities were quantified using HPLC-MS and three antioxidant assays, respectively. For three types of teas, the anticancer effects of their extracts and fractions were assessed using cancer cell lines. The hydrophobic fractions had lower antioxidant capacities than the corresponding hydrophilic fractions, but exhibited superior antiproliferative effects on cancer cells compared with the whole extract and the hydrophilic fraction. Partial least squares-discriminant analysis revealed a strong correlation between the anticancer effects and the theaflavins and flavonols. Therefore, in addition to catechins, the hydrophobic fraction of tea extracts may have beneficial health effects.

## 1. Introduction

Green tea (GT; *Camellia sinensis*) contains large amounts of bioactive polyphenols such as flavan-3-ols (catechins), flavonols, and flavones [1]. Common processed teas include oxidized teas (black tea (BT) and oolong tea), microbial fermented green tea (MT), and post-fermented tea (pu-erh or pu’er tea, also called Chinese fermented dark tea). These representative types of tea contain substantial amounts of phenolic compounds, of which catechins, theaflavins, flavonols, and flavones account for approximately 15%, 4%, 0.4%, and 0.1% of the dry weight (DW), respectively [1,2]. One study simultaneously analyzed over 45 compounds in tea, including catechins and flavonols, using ultra high-performance liquid chromatography (UPLC) coupled with quadrupole time-of-flight (Q-TOF) mass spectrometry (MS) [3]. Recently, 145 compounds such as hydroxycinnamic acids, catechins, and flavonols in GT and pu-erh tea were further characterized using UPLC-Q-Orbitrap-MS/MS in order to discriminate the tea sources by matching the chemical profiles [4].

Advanced analytical instruments and methods, such as high-resolution MS, chemometric analysis, and targeted or non-targeted metabolomics approaches, have provided a wealth of information on the potential health benefits of bioactive phenolics in agricultural plant resources [3,5,6]. Several reports suggested that phenolic compounds in GT and processed teas have anticancer, anti-inflammatory, anti-obesity, and/or antioxidative effects [7,8,9,10]. Many chemometric approaches, such as multivariate analysis, principal component analysis (PCA), linear discriminant analysis, and partial least squares-discriminant analysis (PLS-DA), have been applied to tea phenolics. However, those studies have mainly focused on catechins [11,12], while there exist only a few reports on the correlation between the phenolic composition and function of GT and processed teas. In one such study, multivariate analysis revealed differences between *C. sinensis* and herbal teas [13]. Both the contents and composition of flavonols and flavones also vary among *C. sinensis* cultivars [14]. Additionally, the harvest season, tea manufacturing process, and sample extraction procedure should all be carefully considered when investigating the phenolic profile of teas.

Since the hydrophobic fraction of tea extracts contains very little catechins, we hypothesize that separating the extract into hydrophobic and hydrophilic fractions may allow more comprehensive characterization of the phenolic compounds therein. Chemometrics and multivariate analysis of the different fractions should give less-biased correlations between the phenolic compounds and in vitro biological effects. The whole extracts of green, oxidized, and fermented teas and two fractions of each extract were compared in terms of phenolic profiles and antioxidant and anticancer effects. Many cohort trials have been conducted on the chemopreventive effects of drinking green and fermented teas [15,16,17]. However, we thought that brewing may not sufficiently extract the more hydrophobic compounds, and thus it is important to investigate the impact of different tea fractions.

For better control, green tea and fermented teas were manufactured in this study from the same batch of leaves (i.e., the same cultivar and harvest season). Extracts of these teas were further separated into hydrophilic and hydrophobic fractions, which contain the highest and lowest amounts of catechins, respectively. Those fractions from different teas were then compared in terms of their antioxidant and anticancer effects in vitro.

## 2. Materials and Methods

### 2.1. Chemicals and Reagents

Apigenin, (−)-catechin (C), (−)-catechin gallate (CG), (−)-epicatechin (EC), (−)-epicatechin gallate (ECG), (−)-epigallocatechin (EGC), (−)-epigallocatechin gallate (EGCG), (−)-gallocatechin (GC), (−)-gallocatechin gallate (GCG), theaflavin (TF), theaflavin 3-*O*-gallate (TF3G), theaflavin-3′-*O*-gallate (TF3′G), and theaflavin-3,3′-*O*-digallate (TF3,3′DG) were purchased from FUJIFILM Wako Pure Chemical Industries, Ltd. (Osaka, Japan). The following chemicals and reagents were purchased from Sigma-Aldrich Co., LLC (St. Louis, MO, USA): 2,2-azobis(2-amidinopropane) dihydrochloride (AAPH), 2,2-azino-bis(3-ethylbenzothiazoline-6-sulfonic acid) diammonium salt (ABTS), 3-(4,5-dimethylthiazol-2yl)-2,5-diphenyltetrazolium bromide (MTT), aluminum chloride, ascorbic acid, caffeine, dimethyl sulfoxide (DMSO), Dulbecco’s modified Eagle’s medium (DMEM), 1,1-diphenyl-2-picrylhydrazyl (DPPH), fetal bovine serum (FBS), Folin–Ciocalteu’s phenol reagent, gallic acid (GA), iron(III) chloride hexahydrate (FeCl_3_·6H_2_O), hydrogen peroxide (H_2_O_2_), isoquercitrin (IQ), kaempferol, myricetin, paclitaxel (code: Y0000698), phosphate-buffered saline (PBS), rutin, quercetin, penicillin/streptomycin, Roswell Park Memorial Institute (RPMI) 1640, 2,4,6-Tris(2-pyridyl)-s-triazine (TPTZ), and an in vitro lactate dehydrogenase toxicology assay kit. Formic acid, mass-grade acetonitrile and water, and high-performance liquid chromatography (HPLC)-grade acetonitrile and methanol were purchased from Thermo Fisher Scientific Inc. (Waltham, MA, USA). Water for HPLC was purchased from Burdick & Jackson (Muskegon, MI, USA). All other chemicals were of American Chemical Society grade or higher.

### 2.2. Tea Cultivar and Cell Lines

Fresh tea leaves (*C. sinensis* var. *sinensis*) were harvested from May to June 2017 (2nd harvest of the year) at Osulloc Farm Corp. (Jeju-do, Korea). The leaves were processed as described below to produce five types of tea samples. Leaves of garland chrysanthemum (crown daisy; *Chrysanthemum coronarium* L., for making the co-oxidized green tea) were purchased from Agro-fisheries & Food Corp. (Seoul, Korea). Some of previous research associated with our institute had focused on patients with colorectal adenoma or breast cancer [9,18]. Therefore, in this study the antiproliferative effects were examined using the corresponding cell lines of DLD-1 (colorectal adenocarcinoma cell line; CCL-221™) and E0771 (murine breast cancer cell line; #940001), which were purchased from American Type Culture Collection (Manassas, VA, USA) and CH3 BioSystems LLC (Amherst, NY, USA), respectively.

### 2.3. Preparation of Five Teas

GT was produced from fresh leaves using modern tea factory machines (120K line; Kawasaki Co., Shizuoka, Japan). Briefly, the leaves were harvested with a riding type tea plucking machine, transported to the factory, and placed on automated conveyer belt for loading into the steaming machine. The leaves were in contact with steam for approximately 30 s to deactivate an endogenous enzyme and fix the color. The steamed leaves were rolled and dried until the moisture content was below 4% (*w*/*w*). This GT was used to make MT and natural post-fermented tea (PT) as described below.

BT was prepared by oxidizing the withered leaves in modern tea factory machines (35K line; Kawasaki Co.). Briefly, the fresh tea leaves were naturally withered at 30–40% relative humidity for 18 h, until the moisture content was reduced to 60% (*w*/*w*). A rolling process was conducted for 1 h by applying the Orthodox method [19]. Further oxidation was carried out by spreading the tea leaves on a large wooden board and kept at 40 °C for 7 h, with sparging additional water on the leaf surface. At the end of oxidation process, the leaves were dried in 80 °C air for 2 h. The final moisture content of BT was 4–6% (*w*/*w*).

MT was prepared following previous research with modification [20]. Briefly, dried GT was mixed with 1% (*w*/*v*) sucrose solution and fermented at 50 °C for 72 h after inoculation of *Bacillus subtilis*. Dried MT (5–8% (*w*/*w*) moisture content) was obtained by convective heating without post-maturation.

PT was prepared following the method of Mo et al. [21] with modifications. Briefly, 100 kg of dried GT was mixed with 30 L tap water on a wooden board. The wet GT was collected into one lump on the board, compacted tightly by tapping, and then covered with a thick vinyl film. This allowed natural fungal fermentation to proceed, as indicated by an increase in temperature up to 50 °C. Additional water was supplied after 7 days of fermentation, and then the tea leaves were re-mixed. The fermentation was carried out for a total of three weeks. The fermented tea was dried by heat until the moisture content reached 5% (*w*/*w*).

Co-oxidized GT (CT) was made following a previously reported method [22]. Fresh tea leaves and garland chrysanthemum leaves were washed with water. After removing the excess water, each type of leaves was soaked in liquid nitrogen and crushed into a crude powder. After defrosting, the tea leaves (100 g) and garland chrysanthemum leaves (50 g) were mixed and fermented at 37.5 °C for 3 h using a z-blade mixer (IKA, Staufen, Germany). Then, the fermented mixture was directly used to prepare the extract as described below.

### 2.4. Preparation of Tea Extracts and Fractions

To prepare the extracts of BT, GT, MT, and PT (abbreviated as BTE, GTE, MTE, and PTE, respectively), 50 g of dried tea was ground using an IKA tube mill at 10,000 rpm for 30 s, and then soaked in 10-fold 70% (*v*/*w* of DW) aqueous ethanol at 60 °C for 2 h. To prepare the CT extract (CTE), the fermented mixture was added with 1.75-fold absolute ethanol and soaked at 60 °C for 3 h. Solid particles in the ethanolic extract solutions were removed by a 90-mesh sieve, followed by a 0.22-μm bottle top vacuum filter (Dow Corning, Corning, NY, USA). Ethanol in the extracts was removed using an evaporator (Heidolph Instruments, Schwabach, Germany), and the residue was freeze-dried (Labconco, Kansas City, MO, USA) to produce a powder.

**Figure 1:**
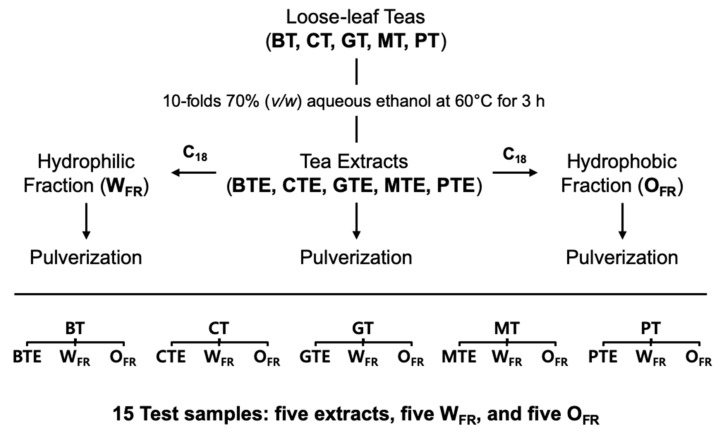
Method of preparing tea extracts and fractions. BT, black tea; CT, co-oxidized green tea, GT, green tea; MT, microbial fermented green tea; PT, post-fermented green tea; BTE, BT extract; CTE, CT extract, GTE, GT extract; MTE, MT extract; and PTE, PT extract. C_18_ means reverse-phase octadecyl-silica column.

Preparative HPLC was used to separate the extract into hydrophilic (W_FR_) and hydrophobic (O_FR_) fractions. Powdered extract (5 g) was dissolved in 50 mL of 70% (*v*/*v*) aqueous ethanol by sonication for 30 min. The mixture was centrifuged to separate the supernatant from the sediment. The sediment was resuspended in 30 mL of 70% (*v*/*v*) ethanol, and the aforementioned liquid extraction was repeated twice. The obtained supernatants were combined and filtered through a 0.45-μm polyvinylidene fluoride syringe filter (Pall Corp., Port Washington, NY, USA). The filtered solution (10 mL) was injected into a preparative liquid chromatography system (ÄKTA Purifier 10; GE Healthcare, Stockholm, Sweden) coupled with a photodiode array detector at wavelengths of 275 nm and 365 nm. The fractions were separated using an octadecyl-silica (ODS) AQ-HG column (120 Å, 10 μm, 20 × 250 mm, column volume (CV) = 78.5 mL; YMC, Kyoto, Japan). Gradient elution was performed with water (solvent A) and acetonitrile (solvent B) at a 10 mL/min of flow rate, and the elution program is described in Figure S1. The eluent from each cycle was divided into W_FR_ and O_FR_ and collected in separate bottles via 12 repeated cycles of injections (Figure S1). After the repeated cycles, 500 mL of 100% acetonitrile was used to elute the remaining compounds in the column, which were combined with the O_FR_. The collected fractions were evaporated and lyophilized as described above for the extracts (Figure 1).

### 2.5. Analysis of Phenolics and Caffeine by HPLC-Ultraviolet (UV)-Coupled Single Quadrupole Mass Detector

Nineteen phenolic compounds and caffeine were quantified according to the following method. Powdered extracts and fractions were dissolved in 10% (*v*/*v*) DMSO in methanol using 20 min of sonication, and then passed through a 0.45-μm GHP syringe filter (Pall Corp.). The filtered samples were injected into an Alliance e2695 HPLC system (Waters Corp., Milford, MA, USA) equipped with an auto-sampler, a quaternary pump, and a Poroshell 120 SB ODS column (120 Å, 2.7 μm, 4.6 × 150 mm; Agilent Technologies, Santa Clara, CA, USA) with an injection volume of 5 μL. The eluent was passed through a Waters Isocratic Solvent Manger simultaneously into a single quadrupole mass detector (MS^S^) and a UV detector, with a retention time gap (0.075 min on average) between the two. The elution program and the conditions for separation and mass detection are described in Supplementary Data I. For the UV detector, gallic acid, catechins, and theaflavins were detected at 275 nm, and flavonols and flavones were monitored at 365 nm. All data were collected and processed using Empower 3 software (Waters Corp.). The relative contents of flavonol glycosides (such as kaempferol, myricetin, and quercetin glycosides) were compared using the peak area of 365 nm.

### 2.6. Post-Data Processing of LC-MS Acquisitions by R Script

To quantify phenolic compounds and caffeine in the standards and samples, the mass chromatograms were smoothed using the mathematical mean method (levels 7−9) by Empower 3 software (Waters Corp.). The two sets of results obtained from UV and MS^S^ were exported into .cvs files. Unnecessary or unmatched UV data with a retention time gap outside the proper range (0.06−0.09 min) were rejected. The matching algorithm was implemented in R software v 4.0.1 (The R Foundation; www.r-project.org) and RStudio Desktop v1.3.959 (RStudio, Boston, MA, USA) based on the same injection identification for MS and UV acquisition (Figure S3 and Supplementary Data I and II).

### 2.7. Determination of Total Flavonoid and Phenolic Contents

The total flavonoid content (TFC) was measured using the method by Kim et al. [23] and expressed in mg catechin equivalents (CE)/g DW of extract (DW_EX_). The total phenolic content (TPC) was determined using a colorimetric method with Folin–Ciocalteu’s phenol reagent [23,24] and presented as mg gallic acid equivalents (GAE)/g DW_EX_. The detailed methods are described in Supplementary Data I.

### 2.8. Measurements of Antioxidant Capacities of Tea Extracts and Fractions

The antioxidant capacities of tea extracts and their fractions were determined by ABTS, DPPH, and ferric reducing antioxidant power (FRAP) assays following the methods described by Kim, Im, Jeong, Jung, Lee, Kim, Park, and Kim [23] and presented as mg vitamin C equivalents (VCE)/g DW_EX_. The detailed methods are described in Supplementary Data I.

### 2.9. Assessment of Antiproliferative Effects of Tea Extracts and Fractions on Cancer Cells

DLD-1 and E0771 cells (5 × 10^3^ cells/well) were incubated in 96-well plates with the sample (10 and 100 mg/L; based on a previous report [10]) for 24 h. Further, paclitaxel, a common anti-cancer drug [25,26], was used for positive control and applied in the same way. After adding MTT, the cells were incubated for another 2 h. Cell viability was confirmed based on the formation of a purple formazan metabolite from MTT. The detailed methods are described in Supplementary Data I.

### 2.10. Multivariate Analysis and Statistical Analysis

PCA was performed using JMP Pro 13 (SAS Institute Inc., Cary, NC, USA) for 20 components in five tea extracts and their fractions (123 rows in total; *n* = 6–9 for each sample). PLS-DA was performed using JMP Pro 13 for three augmented datasets combining the results of TFC, TPC, antioxidant capacities, anticancer effects, and the composition of 20 compounds in five tea extracts and their fractions by matching the sample name column with replications. Dataset (1) contains 612 rows generated from the anticancer effect (80 rows) and compound composition (123 rows). Dataset (2) contains 369 rows generated from the antioxidant capacities (45 rows) and compound composition (123 rows). Dataset (3) contains 240 rows generated from the anticancer effect (80 rows) and antioxidant capacities (45 rows). Nonlinear iterative partial least squares (NIPALS) fit was applied with the fewest factors for which the van der Voet T^2^ significance level exceeds 0.10 [27]. The KFold validation method of PLS-DA was selected with 7 folds.

The data are expressed as mean ± standard error of the mean (*n* = 3). One-way analysis of variance and Tukey–Kramer honestly significant difference test with *p* < 0.05 were implemented in JMP Pro 13.

## 3. Results and Discussion

### 3.1. Phenolic Compositions of Tea Extracts and Fractions

The TFC and TPC in GTE are known to be affected by the extraction method [28]. We used 70% (*v*/*w*) aqueous ethanol to obtain over 30% (*w*/*w*) of the catechins while minimizing the other components such as sugars and proteins [29]. In total, 29 phenolic compounds and caffeine were identified in five teas by HPLC-MS^S^ (Table 1). These identified compounds consist of 10 flavonol glycosides, eight catechins, four theaflavins, three flavonol aglycones, two flavone glycosides, one flavone aglycone, one phenolic acid, and caffeine. Twelve flavonol and flavone glycosides were found in all the five teas, which were prepared from leaves from the same cultivar and harvested in the same season (Table 1 and Figure S2). Compared to GTE, BTE and CTE contained theaflavins that came from the intrinsic enzymatic action of catechin polymerization (Figure S2A,B). MTE and PTE of microbial fermented teas contained higher amounts of gallic acid and non-epicatechins (i.e., C, CG, GC, and GCG), and a lower amount of flavonol aglycones compared with GTE (Figure S2D,E). Except for CT (which contains about 1/3 crown daisy leaves in fresh weight and therefore less caffeine), extracts of the other four teas had similar levels of caffeine (approximately 70 mg/g DW_EX_). Meanwhile, the extract of crown daisy leaves showed no detectable signals at the wavelength of UV 275 and 365 nm (data not shown).

**Figure 2:**
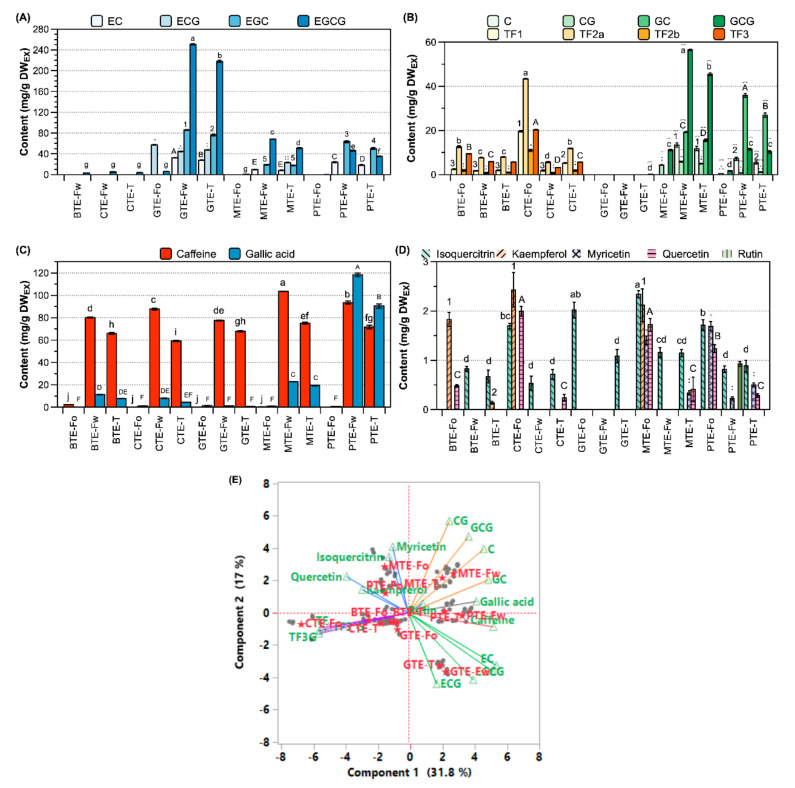
Contents of phenolic compounds and caffeine in the five tea extracts and their fractions. (**A**) Epicatechins, (**B**) theaflavins and non-epicatechins, (**C**) caffeine and gallic acid, (**D**) flavonol glycosides, and (**E**) Correlation between 20 identified compounds and the extracts and fractions by PCA. Different letters, numbers, and dotted marks on the bars indicate significant differences according to the Tukey–Kramer honestly significant difference test (*p* < 0.05). The suffixes -T, -Fw, and -Fo on the X-axis indicate whole extract, hydrophilic fraction (W_FR_), and hydrophobic fraction (O_FR_), respectively. DW_EX_, dry weight of extract; EC, (−)-epicatechin; ECG, (−)-epicatechin gallate; EGC, (−)-epigallocatechin; EGCG, (−)-epigallocatechin gallate; C, (−)-catechin; CG, (−)-catechin gallate; GC, (−)-gallocatechin; GCG, (−)-gallocatechin gallate; TF1, theaflavin; TF2a, theaflavin 3-*O*-gallate; TF2b, theaflavin-3′-*O*-gallate; and TF3, theaflavin-3,3′-*O*-digallate.

In detail, the levels (unit: mg/g DW_EX_) of EGCG and EGC were 218 and 76 for GTE, 51 and 18 for MTE, 35 and 50 for PTE, below 5 of them for BTE, and none for CTE, respectively (Figure 2A and Table S1). In GTE, MTE, and PTE, the levels of ECG and EC were approximately 2–5 folds lower than those of EGCG and EGC, and their relative proportions (ECG/EC and EGCG/EGC) were similar. Meanwhile, ECG and EC were not detected in BTE and CTE. The total theaflavins were 16.6 and 24.6 mg/g DW_EX_ for BTE and CTE, respectively. The levels of TF and TF3G were higher in CTE than in BTE, while these two extracts have similar contents of TF3′G and TF3,3′DG (Figure 2B and Table S1). Substantial amounts of non-epicatechins were present in MTE and PTE. The total amount of non-epicatechins was 77.9 and 55.4 mg/g DW_EX_ for MTE and PTE, respectively (Figure 2B and Table S1). The least amount of gallic acid (<1 mg/g DW_EX_) was found in GTE, followed by BTE and CTE (4.5–7.9), and then MTE and PTE (20–90) according to Figure 2C and Table S1. The content of caffeine was 68 mg/g DW_EX_ for GTE, and 60–75 mg/g DW_EX_ for the four processed tea extracts (Figure 2C and Table S1). As a representative flavonol glycoside, isoquercitrin (quercetin-3-*O*-glucoside) was present at ~0.7 mg/g DW_EX_ in BTE and CTE and at ~1.0 mg/g DW_EX_ in GTE, MTE, and PTE. Meanwhile, rutin (quercetin-3-*O*-rutinoside) was detected in all five tea extracts (Figure 2D and Table S1). Quercetin aglycone was not detected in BTE and GTE, while it was detected at 0.3–0.4 mg/g DW_EX_ in CTE, MTE, and PTE. Kaempferol was detected only in BTE (Table S1).

Each tea extract was divided in two fractions in this study, and the yields (*w*/*w*) were approximately 0.75 for W_FR_ and 0.15 for O_FR_. In each extract, the 20 identified compounds (numbered peaks in Table 1 and quantified in Figure 2) had the following distribution in the two fractions: hydrophilic compounds (e.g., catechins and gallic acid), 1.2–1.3 folds in W_FR_ and <0.05-fold in O_FR_; amphiphilic compounds (e.g., rutin and isoquercitrin), 1.2–2.4 folds in O_FR_ and 0.8–1.0 folds in W_FR_; hydrophobic compounds, 3.4–8.3 folds in O_FR_ and none in W_FR_ for e.g., myricetin and quercetin as well as 1.3–6.1 folds in O_FR_ and 0.4–1.0 folds in W_FR_ for e.g., theaflavins (data not shown). Taken together, the composition of the 20 compounds was different among the five tea extracts and their fractions, and these differences were well distinguished for the 15 samples by PCA as shown in Figure 2E.

The relative content of 10 flavonol glycosides in the four processed tea extracts differed from that in GTE (Table S2). Myricetin glycosides completely disappeared in BTE and CTE, presumably due to intrinsic enzymatic reaction according to a previous report [31]. The contents of apigenin glycoside (peak g), kaempferol glycoside (peak j), and quercetin glycoside (peak f) in MTE and PTE were increased because of their microbial transformation by detaching the monoglycosyl (arabinose or glucose) moieties of peak a, peak i, and peak d or e, respectively. Those changes are to be elucidated in further studies, while the presence of apigenin glycoside in pu-erh tea has been reported [32].

**Figure 3:**
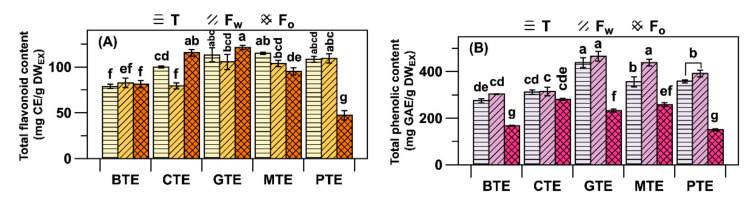
Contents of (**A**) total flavonoids and (**B**) total phenolics of five tea extracts and their fractions. Labels T, Fw, and Fo on top of the graphs indicate whole extract, W_FR_, and O_FR_, respectively. Lowercase letters on the bars indicate significant differences according to the Tukey–Kramer honestly significant difference test (*p* < 0.05). CE, catechin equivalents; and GAE, gallic acid equivalents.

The TPC was similar across the five tea extracts (~100 mg GAE/g DW_EX_) except for BTE. TFC is ranked as GTE (440 mg CE/g DW_EX_) > MTE and PTE (~350) > BTE and CTE (~300), as shown in Figure 3A,B. The proportion of TFC increased in the O_FR_ of CTE and GTE, while in the other three extracts its proportion increased in the W_FR_ (Figure 3 and Table S3). The proportion of TPC in each extract changed to 0.64 in O_FR_ and 1.10 in W_FR_ on average (data not shown).

Compared to the other extracts, EGCG was more enriched in GTE and GCG was more enriched in MTE (Figure 2A,B). MTE and PTE had higher portions of non-epicatechins, due to their chemical instability in the aqueous or highly humid conditions during microbial fermentation [33]. Catechins are unstable in aqueous conditions by undergoing epimerization or ring fission, depending on environmental factors such as the moisture and pH [34]. Despite those changes in catechins, the TFC values in GTE, MTE, and PTE were not statistically different (Figure 3). In contrast, TFC and TPC were significantly (*p* < 0.05) reduced in BTE and CTE compared to those in GTE, due to the disappearance of catechins and the formation of theaflavins (Figure 3B) [35]. A distinctive increase in gallic acid in PTE (and to a lesser extent in MTE) was due to the fungal and bacterial enzymatic degalloylation of gallated catechins [4]. The least amount of gallic acid was found in BTE and CTE, owing to the action of intrinsic and extrinsic plant enzymes such as polyphenol oxidase and peroxidase [36].

### 3.2. Antioxidant Capacities of Tea Extracts and Fractions

**Figure 4:**
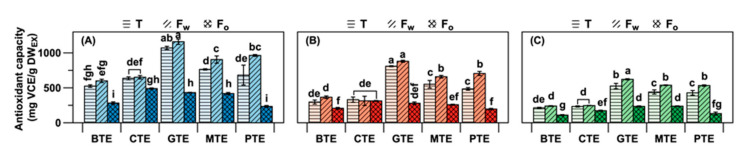
Antioxidant capacities of five tea extracts and their fractions. (**A**) ABTS, (**B**) DPPH, and (**C**) FRAP. Legends: labels T, Fw, and Fo on the graph indicate whole extract, W_FR_, and O_FR_, respectively. Lowercase letters a–g on the bars indicate significant differences according to the Tukey–Kramer honestly significant difference test (*p* < 0.05). VCE stands for vitamin C equivalents.

The antioxidant capacities of GTE were 1071, 810, and 526 mg VCE/g DW_EX_ when measured using the ABTS, DPPH, and FRAP assays, respectively (Figure 4 and Table S3). The antioxidant capacities measured in the ABTS assay for the other four extracts were only approximately 50–70% those of GTE. When measured in the DPPH and FRAP assays, the antioxidant capacities of BTE and CTE decreased to approximately 40% those of GTE, and the antioxidant capacities of MTE and PTE reduced to approximately 80% those of GTE. Recall that the TFC and TPC in each tea extract became lower in O_FR_ and higher in W_FR_. In all three assays, the O_FR_ of BTE and CTE had approximately 50–75% of antioxidant capacities of the corresponding extract, while the O_FR_ of the other three extracts retained approximately 30–50% of the antioxidant capacities (Figure 4A–C; Table S3). The W_FR_ of PTE had approximately 120–150% of antioxidant capacities in the ABTS, DPPH, FRAP assays compared to the corresponding extract, while W_FR_ of the other four extracts had approximately 100–120% of the antioxidant capacities. The antioxidant capacities of teas are primarily associated with monomeric flavan-3-ols (catechins) [37]. Generally, the antioxidant capacity of flavonol glycosides decreases as the number of conjugated glycosides increases, owing to the masking effect of complex glycoside structures [38]. The decreased antioxidant capacities of O_FR_ could be explained by assuming that the number of free hydroxyl (−OH) group at C3 position in flavonoids is crucial for maintaining antioxidant capacity [39]. In this context, a higher content of flavonol glycosides and a lower amount of catechins contributed to the low antioxidant capacity of O_FR_ [10,40].

### 3.3. Antiproliferative Effects of Tea Extracts and Fractions on Cancer Cells

**Figure 5:**
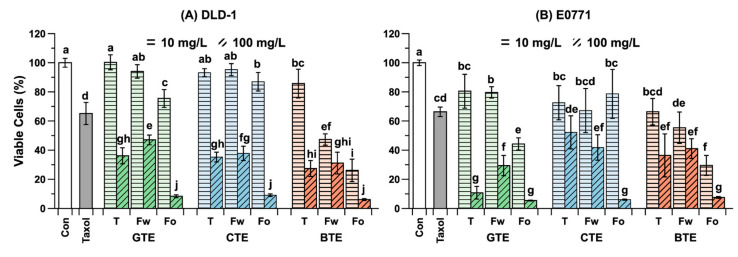
Antiproliferative effects of three tea extracts (GTE, CTE, and BTE) and their fractions. (**A**) Viable DLD-1 cells after 24 h and (**B**) Viable E0771 cells after 24 h. Con = negative control (buffer treatment), and Taxol = positive control (paclitaxel, 10 nM). T, Fw, and Fo on the X-axis mean whole extract, W_FR_, and O_FR_, respectively. Different letters a–j on the bars indicate significant differences according to the Tukey–Kramer honestly significant difference test (*p* < 0.05).

The anticancer effects of GTE, CTE, and BTE as well as their two fractions were examined using two adenoma cell lines (DLD-1 and E0771). A chemotherapeutic agent (paclitaxel) was used as positive control, and buffer was used for negative control. At concentration of 10 μg/mL, these three extracts did not affect the viability of DLD-1 cells but reduced that of E0771 cells to 70–80% of negative control. Meanwhile, paclitaxel reduced the viability of both types of cells to ~70% (Figure 5). At 100 μg/mL, GTE and BTE significantly (*p* < 0.05) reduced the viability of DLD-1 and E0771 cells to 30–40% and 10–50% that of the negative control, respectively. Therefore, tea extracts at a sufficiently high concentration have anticancer effects comparable to chemotherapy drugs. Among the fractions, only the W_FR_ and O_FR_ of BTE exhibited anticancer effects at 10 μg/mL for DLD-1 compared with the positive control, and the O_FR_ of GTE and BTE for E0771. At 100 μg/mL, all W_FR_ and O_FR_ of the three extracts exhibited significant (*p* < 0.05) anticancer effects on the two types of adenoma cells compared with the positive control. Overall, the anticancer effects are ranked as O_FR_ (<9% viability) > extract (27–36%) ≥ W_FR_ (30–47%). Specifically, all three W_FR_ had equal or less effects compared to the corresponding whole extract. These anticancer effects of the tea extracts are consistent with the previously reported inhibitory effects of tea constitutes on various adenoma cells [41]. Furthermore, O_FR_ showed significantly stronger anticancer effects than those offered by the corresponding W_FR_. Thus, in addition to the catechins, minor compounds in O_FR_ such as flavonols and theaflavins have crucial biological effects [42,43].

### 3.4. Multivariate Analysis of Phenolic Composition, Antioxidant Capacities, and Anticancer Effects of Tea Extracts and Fractions

**Figure 6:**
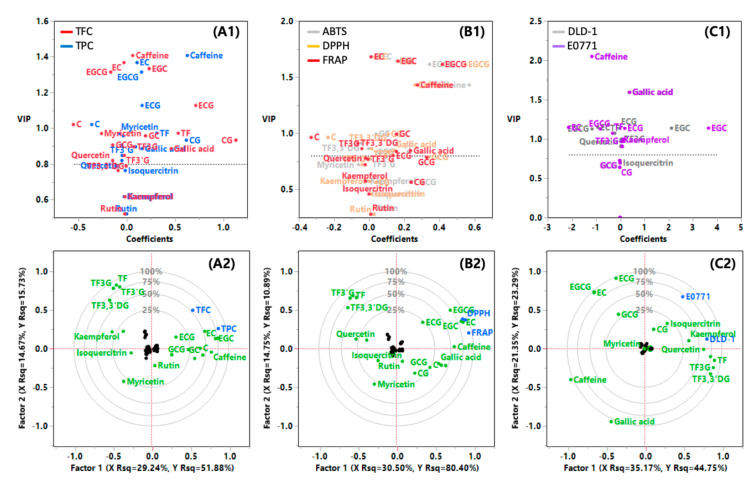
Partial least squares-discriminant analysis for the 20 compounds identified in five tea extracts and their biological effects. (**A**) TFC/TPC, (**B**) antioxidant capacities, and (**C**) anticancer effects. (**1**): VIP versus coefficients matrix diagram for centered and scaled data and (**2**) correlation loading plots. Factor 1 and factor 2 are constructed to account for the correlation or covariance between the observed variables. Factor rotation was used to change the reference axes of the factors to increase their interpretability.

Our chemometric analysis used PLS-DA to distinguish which compounds are correlated with the TFC and TPC, as well as their contribution to the measured biological effects. The variable importance in projection (VIP) method was adopted to screen the influential variables with the criterion of VIP value > 0.8. A diagram of VIP versus coefficients was generated for the centered and scaled data, highlighting key compounds that contribute to the TFC, TPC, antioxidant capacities, and anticancer effects (Figure 6A1,B1,C1). Then, the corresponding correlation loading plots were created for the 20 compounds (Figure 6A2,B2,C2). The established models of PLS-DA were built using a valid number of factors (10−15) that responded to the lowest root mean PRESS values (data not shown).

Four epicatechins (EC, ECG, EGC, and EGCG) were major contributors to TFC and TPC with high VIP values (>1.0), and were more strongly correlated to TPC than TFC (Figure 6A1,A2). The good quality of the fit was confirmed by the parameters of fitness (cumulative *R*^2^*X* = 0.996 and *R*^2^*Y* = 0.896) and predictability (*Q*^2^ = 1.000) [44]. This result indicates that the contents of these four epicatechins can represent the TPC in tea extracts and fractions with statistical significance (Table S4). The PLS-DA for TFC and TPC explained 44% of *X* variance and 68% of *Y* variance from the sum of factor 1 and factor 2 (Figure 6A2). Isoquercitrin and kaempferol are considered to be the least correlated to the TFC and TPC of tea extracts and fractions. In a similar manner, four epicatechins were highly correlated to the three assay of antioxidant capacities with high VIP values (>1.5) and large coefficients. The fit had good quality in terms of the cumulative *R*^2^*X* (0.996), *R*^2^*Y* (0.970), and *Q*^2^ (1.000). The antioxidant capacities were significantly and strongly correlated to the content of these four epicatechins in tea extracts and fractions (Figure 6B1,B2 and Table S4). The PLS-DA for antioxidant capacities explained 45% of *X* variance and 91% of *Y* variance from the sum of factor 1 and factor 2 (Figure 6B2). On the other hand, caffeine and gallic acid showed high VIP with moderate coefficient values, and EGC showed moderate VIP with large coefficient for the anticancer effects. The viability of DLD-1 cells was mainly influenced by theaflavins and flavonols, whereas that of E0771 cells was mainly influenced by catechins (Figure 6C1,C2). The PLS-DA for anticancer effects explained 57% of *X* variance and 68% of *Y* variance from the sum of factor 1 and factor 2 (Figure 6C2). Multivariate analysis indicates that the anticancer effects of tea extracts and fractions are more correlated to the contents of flavonols and theaflavins than those of catechins (Table S5 and Figure S4). Caffeine was negatively correlated to the anticancer effects. In some cases, care is required to properly apply multivariate analysis to the in vitro function of bioactive compounds [45,46]. Our PLS-DA gave meaningful correlation coefficients for the anticancer effect of phenolic compounds.

## 4. Conclusions

This study explored the phenolic compositions of extracts from five authentic teas (one green tea, two oxidized teas, and two microbial fermented teas) and their hydrophilic and hydrophobic fractions. These phenolic profiles were further correlated to the antioxidant capacities. PCA revealed that the 20 identified compounds were well distributed across the 15 tea extracts and fractions. Especially, TPC may be more useful than TFC for estimating the antioxidant capacities. Interestingly, the hydrophobic fractions of tea extracts exhibited stronger antiproliferative effects on both colorectal and breast carcinoma cell lines than their hydrophilic counterparts. Among the hydrophobic compounds, multivariate analysis additionally revealed that flavonols and theaflavins may be important indicators of the biological effects. Further research is needed to elucidate the mechanisms behind the observed anticancer effects. However, considering that most research conducted thus far has focused on catechins, our findings suggest that core compounds in the hydrophobic fraction of teas should also be explored. Moreover, when studying the biological effects of other edible plant sources, such as various teas, coffees, and herbs, there is a similar need to screen all the components and not just the major ones.

## Figures and Tables

**Table 1:** Identification of compounds in the five tea extracts.

Peak ^a^	Class ^b^	RT_av_^c^	Molecular Mass ^d^	𝜆_max1_/𝜆_max2_^e^	Formula	Identification ^f^
1	PA	3.55	170.02	270.7/-	C_7_H_6_O_5_	Gallic acid
2	F3	5.53	306.07	269.5/-	C_15_H_14_O_7_	(−)-Gallocatechin
3	F3	7.38	306.07	269.5/-	C_15_H_14_O_7_	(−)-Epigallocatechin
4	F3	8.06	290.08	279.0/-	C_15_H_14_O_6_	(−)-Catechin
5	MX	8.61	194.08	273.0/-	C_8_H_10_N_4_O_2_	Caffeine
6	F3	9.67	290.08	279.0/-	C_15_H_14_O_6_	(−)-Epicatechin
7	F3	10.16	458.08	274.2/-	C_22_H_18_O_11_	(−)-Epigallocatechin gallate
8	F3	11.41	458.08	274.2/-	C_22_H_18_O_11_	(−)-Gallocatechin gallate
a	FE	11.52	563.14	270.7/333.8	C_32_H_27_O_14_	Apigenin-6-*C*-glucosyl-8-*C*-arabinoside
b	FL	12.65	479.08	258.8/355.3	C_21_H_19_O_13_	Myricetin-3-*O*-galactoside
c	FL	13.13	479.08	255.2/358.9	C_21_H_19_O_13_	Myricetin-3-*O*-glucoside
d	FL	13.88	771.20	255.2/354.1	C_33_H_39_O_21_	Quercetin-3-*O*-galactosylrutinoside
e	FL	15.18	771.20	255.2/352.9	C_33_H_39_O_21_	Quercetin-3-*O*-glucosylrutinoside
f	FL	16.42	609.15	268.3/338.6	C_27_H_29_O_16_	Quercetin-3-*O*-rhamnosylgalactoside
9	FL	16.87	609.15	255.2/352.9	C_27_H_29_O_16_	Quercetin-3-*O*-rhamnosylglucoside
10	F3	17.18	442.09	276.6/-	C_22_H_18_O_10_	(−)-Epicatechin gallate
g	FE	17.21	431.10	245.8/344.6	C_26_H_19_O_10_	Apigenin-6-*C*-glucoside or isomer
h	FL	17.77	463.09	255.2/355.3	C_21_H_19_O_12_	Quercetin-3-*O*-galactoside
11	FL	17.91	463.09	255.2/352.9	C_21_H_19_O_12_	Quercetin-3-*O*-glucoside
12	F3	18.18	442.09	276.6/-	C_22_H_18_O_10_	(−)-Catechin gallate
i	FL	18.74	755.20	264.7/346.9	C_33_H_39_O_20_	Kaempferol-3-*O*-glucosylrutinoside
j	FL	20.43	593.15	264.7/346.9	C_27_H_29_O_15_	Kaempferol-3-*O*-rhamnosylglucoside
13	FL	24.30	317.03	254.1/376.7	C_15_H_9_O_8_	Myricetin
14	FL	32.05	563.13	268.3/375.5	C_29_H_24_O_12_	Theaflavin
15	FL	32.65	301.03	254.1/363.9	C_15_H_9_O_7_	Quercetin
16	TF	35.28	704.17	270.7/375.5	C_36_H_32_O_15_	Theaflavin-3-*O*-gallate
17	TF	37.20	704.17	274.2/375.5	C_36_H_32_O_15_	Theaflavin-3′-*O*-gallate
18	TF	37.75	868.15	274.2/375.5	C_43_H_32_O_20_	Theaflavin-3,3′-*O*-digallate
19	FE	42.08	270.05	265.9/337.4	C_15_H_10_O_5_	Apigenin
20	FL	43.41	285.04	264.7/363.9	C_15_H_9_O_6_	Kaempferol

^a^ Lowercase characters indicate flavonol and flavone glycosides which were not included in quantitative analysis. ^b^ Identification inferred from the literature [10,30]. F3, flavan-3-ol; FE, flavone; FL, flavonol; MX, methylxanthine; PA, phenolic acid; TF, theaflavin. ^c^ RT_av_: average retention time of UV detection. ^d^ All acquisitions were carried out in the negative mode (*m*/*z*, [M − H]^−^), except for caffeine which used the positive mode (*m*/*z*, [M + H]^+^). ^e^ 𝜆_max1_/𝜆_max2_: obtained by other HPLC coupled with photodiode array detector (Waters Corp.) with same separation methods as described in this article (solvent: 0.1% (*v*/*v*) formic acid in water and acetonitrile; pH ≈ 2.7). ^f^ Refer to previous report for identification of FL and FE [10].

## Supplementary Materials

The following is available online at https://www.mdpi.com/2076-3921/9/10/1015/s1, Supplementary Data I: Details of methods, preparative chromatography, HPLC traces of five tea extracts, phenolic content of five tea extracts and fractions. Figure S1. Preparative separation of five tea extracts. Figure S2. HPLC chromatograms of various teas. Figure S3. Process flow for collection of valid UV data. Figure S4. Correlation loading plot of PLS-DA for anticancer effect by antioxidant capacities, and total flavonoid and phenolic contents. Table S1. Phenolic content of the five tea extracts and fractions. Table S2. Flavonol and flavone glycoside contents of the five tea extracts. Table S3. Total flavonoid and phenolic contents, and antioxidant capacities of the five tea extracts and their fractions. Table S4. Correlation result of multivariate analysis among total flavonoid and phenolic contents, antioxidant capacities, and phenolic compounds in five tea extracts and their fractions. Table S5. Correlation result of multivariate analysis for the anticancer effects, antioxidant capacity, and phenolic compounds of three tea extracts and their fractions. Script 1. Coded script for collecting valid UV data based on MS data. Supplementary Data II: Quantitative raw data of five tea extracts and fractions by HPLC-UV-MS^S^ for 20 compounds, R coding script for obtaining valid UV data based on MS data, and processed valid result data by the R coding script (Files: Tea_MS1.csv, Tea_MS2.csv, Tea_UV_275.csv, Tea_UV_365.csv, 5Teas.R, and 5Tea_results.csv).
