# Supplementary material for: Chemometric Analysis of Extracts and Fractions from Green, Oxidized, and Microbial Fermented Teas and Their Correlation to Potential Antioxidant and Anticancer Effects
Source: Antioxidants (Basel). 2020 Oct 19;9(10):1015. doi: 10.3390/antiox9101015 (PMC7650543; doi:10.3390/antiox9101015)
Supplement: Supplementary file 1 [file antioxidants-09-01015-s001.zip › Suppl/antioxidants-954153-(S)-proof.docx]

Supplementary material I

Chemometric Analysis of Extracts and Fractions from Green, Oxidized, and Microbial Fermented Teas and Their Correlation to Potential Antioxidant and Anticancer Effects

Chan-Su Rha ^1,^*, Young Sung Jung ^2^, Jung-Dae Lee ^3^, Davin Jang ^2^, Mi-Seon Kim ^2^, Min-Seuk Lee ^3^, Yong Deok Hong ^1^ and Dae-Ok Kim ^2,^*

^1^ AMOREPACIFIC R&D Center, Yongin 17074, Republic of Korea; hydhong@amorepacific.com (Y.D.H)

^2^ Department of Food Science and Biotechnology, Kyung Hee University, Yongin 17104, Republic of Korea; chembio@khu.ac.kr (Y.S.J), davin1031@khu.ac.kr (D.J), miseonkim95@khu.ac.kr (M.-S. K), DOKIM05@khu.ac.kr; (D.-O.K.)

^3^ Osulloc Tea R&D Center, Osulloc Farm Corporation, Seogwipo 63521, Republic of Korea; jedlee@osullocfarm.com (J.-D.L), leems@osullocfarm.com (M.-S.L)

***** Correspondence: teaman@amorepacific.com (C.-S.R.); DOKIM05@khu.ac.kr (D.-O.K.); Tel.: +82-31-280-5981 (C.-S.R.), Tel.: +82-31-201-3796 (D.-O.K.)

Received: 18 September 2020; Accepted: 16 October 2020; Published: date

**Analysis of phenolics and caffeine by HPLC-UV-coupled single quadrupole mass detector**

The column temperature was maintained at 30 °C. The mobile phases were 0.1% (*v/v*) formic acid in water (solvent A) and 0.1% (*v/v*) formic acid in acetonitrile (solvent B), and the flow rate was 0.8 mL/min. All solvents used were filtered and degassed. The linear gradient was as follows: 92% A/8% B at 0 min, 92% A/8% B at 2 min, 88% A/12% B at 3 min, 84% A/16% B at 4 min, 84% A/16% B at 12 min, 80% A/20% B at 15 min, 80% A/20% B at 18 min, 76% A/24% B at 22 min, 74% A/26% B at 32 min, 72% A/28% B at 35 min, 72% A/28% B at 43 min, 40% A/60% B at 46 min, 92% A/8% B at 47 min, and 92% A/8% B at 50 min.

Single quadrupole mass detection was performed using the following parameters: capillary voltage, 0.8 kV; probe temperature, 600 °C; electrospray ionization source temperature, 120 °C; and desolvation nitrogen gas pressure, 90 psi. Cone voltages were allocated according to the chemical being analyzed: caffeine, 5 V; gallic acid and catechins, 10 V; others, 15 V. Single ion recording was performed in positive mode for caffeine and negative mode for phenolics.

**Determination of total phenolic and flavonoid contents**

For measurements of total phenolic content (TPC), 10 mg of catechin, gallic acid, and tea extract were dissolved in 1 mL of DMSO, and then diluted with deionized water. Tea extract (200 μL) were mixed with deionized water (2.6 mL). Then, 200 μL of FC reagent was added to each mixture at 0 min. Two milliliters of 7% (*w/v*) sodium carbonate solution was added at 6 min. At 90 min, the absorbance of mixture was measured at 750 nm using a spectrophotometer (SPECTRONIC 200). The TPC of each extract was presented as mg gallic acid equivalents (GAE)/g DW of tea extract.

For measurements of total flavonoid content (TFC), diluted tea extract (500 μL) was mixed with deionized water (3.2 mL), and then 5% (*w/v*) NaNO_2_ (150 μL) was added. Then, 10% (*w/v*) AlCl_3_ (150 μL) was added at 5 min and 1N NaOH (1.0 mL) was added at 6 min. The absorbance was measured at 510 nm using a spectrophotometer (SPECTRONIC 200; Thermo Fisher Scientific Inc., Waltham, MA, USA). The TFC of each extract was expressed as mg catechin equivalents (CE)/g DW of tea extract.

**Measurements of Antioxidant Capacities of Tea Extracts and Fractions**

In ABTS assay, the ABTS radical solution (in PBS) was adjusted to an absorbance of 0.650 ± 0.020 at 734 nm. The reaction between ABTS radical solution (980 μL) and tea extracts (20 μL) were reacted at 37 °C for 10 min. The absorbance was measured at 734 nm using a spectrophotometer (SPECTRONIC 200).

In DPPH assay, the absorbance of DPPH radical solution (in methanol) was set to 0.650 ± 0.020 at 517 nm. The reaction between DPPH radical solution (2.95 mL) and tea extracts (50 μL) were reacted at ambient temperature for 10 min. The absorbance was measured at 517 nm using a spectrophotometer (SPECTRONIC 200).

In FRAP assay, the FRAP reagent solution was prepared by mixing 0.3 M sodium acetate buffer solution (pH 3.6), 10 mM TPTZ in 40 mM HCl, and 20 mM FeCl_3_·6H_2_O at a ratio of 10:1:1 [1]. The reactions between the FRAP reagent solution (950 μL) and diluted tea extract (50 µL) was reacted at ambient temperature for 30 min, and then the absorbance was measured at 593 nm using spectrophotometer (SPECTRONIC 200). The antioxidant capacity of each extract measured using these three antioxidant assays was expressed as mg vitamin C equivalents (VCE)/g DW of tea extract.

**Assessment of Anticancer Effects of Extracts and Fractions**

DLD-1 cells were cultured in DMEM supplemented with 10% FBS and 0.1% penicillin/streptomycin solution in a humidified incubator at 37 °C with 5% CO_2_. E0771 cells were grown in RPMI-1640 supplemented with 10% FBS and 0.1% penicillin/streptomycin and were cultured under the same temperature and humidity conditions. Purple formazan crystals were dissolved in DMSO and absorbance was measured at a wavelength of 570 nm using a microplate reader (Infinite M200 Pro; Tecan, Männedorf, Switzerland). The results are represented as the percent of absorbance relative to that of the control cells.

**Figure** **S1**. Preparative separation of five tea extracts. (A) Black tea extract (BTE), (B) co-oxidized tea extract (CTE), (C) green tea extract (GTE), (D) microbial fermented tea extract (MTE), and (E) post-fermented tea extract (PTE). Line colors indicate as follows: blue, absorbance at UV 275nm; red, absorbance at UV 365 nm; and green, solvent B%. F3 and F4 are corresponded to hydrophilic and hydrophobic fractions, respectively. Elution program: 0.45 column volume (CV), 84% A/16% B; 1.80 CV, 84–80% A/16–20% B; 0.45 CV, 80% A/20% B; 0.45 CV, 80–78% A/20–22% B; 0.45 CV, 78–20% A/22–80% B; 0.45 CV, 20–0% A/80–100% B; 0.45 CV, 0–100% A/100–0% B; and 0.45 CV, 100% A/0% B.


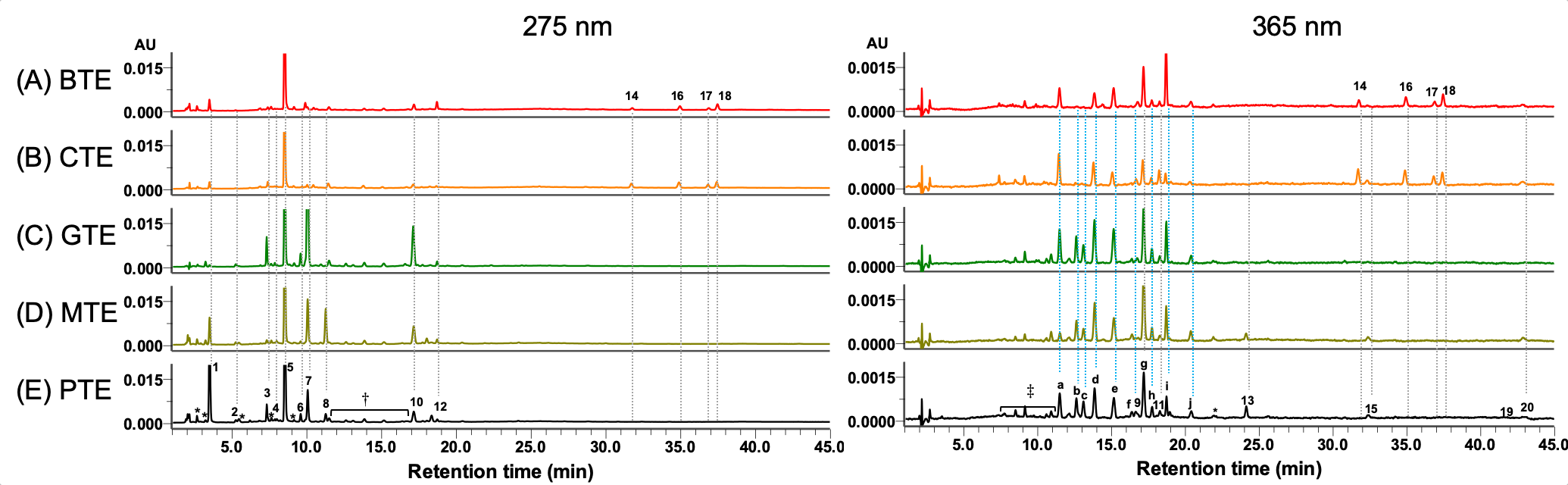


**Figure** **S2**. HPLC chromatograms of (A) BTE, (B) CTE, (C) GTE, (D) MTE, and (E) PTE detected by UV at 275 nm and 365 nm. Refer to **Table 1** for the identification of each numbered peak. Lowercase characters on the graph indicate unquantified flavonol and flavone glycosides. The marks † and ‡ indicate flavonol/flavone and flavan-3-ol peaks at 257 nm and 365 nm, respectively, and * indicates unidentified peaks. All tea extracts were analyzed at 200 mg/L concentration. Vertical gray and blue lines indicate known (by standard compound) and presumed (by tandem mass) compounds, respectively.

**Table** **S1**. Phenolic content of the five tea extracts and fractions

|  | BTE ^a^ |  |  | CTE ^a^ |  |  | GTE ^a^ |  |  | MTE ^a^ |  |  | PTE ^a^ |  |  |
| --- | --- | --- | --- | --- | --- | --- | --- | --- | --- | --- | --- | --- | --- | --- | --- |
| Compound ^b^  (unit: mg/g DW_EX_) | T ^c^ | Fw ^c^ | Fo ^c^ | T | Fw | Fo | T | Fw | Fo | T | Fw | Fo | T | Fw | Fo |
| C | **n.d. ^d^** | **n.d.** | **n.d.** | **n.d.** | **n.d.** | **n.d.** | **n.d.** | **n.d.** | **n.d.** | **11.81 ± 2.77ae** | **13.37 ± 2.91a** | **n.d.** | **5.49 ± 2.16b** | **7.22 ± 2.14b** | **n.d.** |
| Caffeine | **66.03 ± 2.57h** | **80.13 ± 1.62d** | **2.32 ± 0.22j** | **59.11 ± 1.69i** | **87.75 ± 2.25c** | **0.22 ± 0.13j** | **67.91 ± 2.02gh** | **77.54 ± 1.02de** | **0.10 ± 0.01j** | **75.18 ± 2.68ef** | **103.50 ± 0.55a** | **0.45 ± 0.06j** | **71.65 ± 4.81fg** | **93.51 ± 4.03b** | **n.d.** |
| CG | **n.d.** | **n.d.** | **n.d.** | **n.d.** | **n.d.** | **n.d.** | **<LOD** | **n.d.** | **<LOD** | **4.98 ± 0.63b** | **5.86 ± 0.67a** | **4.32 ± 0.47b** | **1.14 ± 0.41c** | **0.82 ± 0.14c** | **0.56 ± 0.02c** |
| EC | **<LOD ^e^** | **<LOD** | **n.d.** | **n.d.** | **n.d.** | **n.d.** | **27.72 ± 1.73b** | **32.49 ± 0.95a** | **n.d.** | **8.13 ± 2.64e** | **9.70 ± 1.34e** | **n.d.** | **18.50 ± 3.14d** | **23.72 ± 3.26c** | **n.d.** |
| ECG | **n.d.** | **n.d.** | **n.d.** | **n.d.** | **n.d.** | **n.d.** | **47.43 ± 1.94b** | **44.66 ± 1.25c** | **57.38 ± 1.11a** | **23.23 ± 0.75d** | **n.d.** | **n.d.** | **<LOD** | **<LOD** | **n.d.** |
| EGC | **n.d.** | **n.d.** | **n.d.** | **n.d.** | **n.d.** | **n.d.** | **76.14 ± 4.85b** | **85.88 ± 1.79a** | **n.d.** | **17.68 ± 2.25e** | **19.08 ± 2.24e** | **n.d.** | **50.21 ± 5.70d** | **63.41 ± 6.08c** | **n.d.** |
| EGCG | **LL** | **2.72 ± 0.03g** | **n.d.** | **3.74 ± 0.82g** | **5.24 ± 1.79g** | **n.d.** | **218.15 ± 6.80b** | **250.92 ± 3.05a** | **6.01 ± 0.38g** | **51.02 ± 2.71d** | **68.02 ± 0.85c** | **0.74 ± 0.18g** | **35.12 ± 2.84f** | **46.25 ± 2.59e** | **0.20 ± 0.00g** |
| Gallic acid | **7.87 ± 0.42de** | **11.32 ± 0.50d** | **0.35 ± 0.02f** | **4.50 ± 0.30ef** | **8.04 ± 0.39de** | **1.08 ± 0.08f** | **0.90 ± 0.10f** | **1.21 ± 0.15f** | **1.49 ± 0.25f** | **19.55 ± 0.85c** | **22.76 ± 0.30c** | **1.01 ± 0.13f** | **90.48 ± 5.76b** | **118.42 ± 4.69a** | **0.59 ± 0.15f** |
| GC | **n.d.** | **n.d.** | **n.d.** | **n.d.** | **n.d.** | **n.d.** | **n.d.** | **n.d.** | **n.d.** | **15.56 ± 1.67d** | **19.23 ± 0.73c** | **n.d.** | **26.93 ± 3.12b** | **35.87 ± 2.72a** | **n.d.** |
| GCG | **n.d.** | **n.d.** | **n.d.** | **n.d.** | **n.d.** | **n.d.** | **0.24 ± 0.00d** | **<LOD** | **n.d.** | **45.56 ± 1.83b** | **56.47 ± 1.13a** | **11.13 ± 1.18c** | **10.30 ± 2.03c** | **11.53 ± 1.03c** | **1.74 ± 0.44d** |
| Isoquercitrin | **0.67 ± 0.26d** | **0.83 ± 0.09d** | **n.d.** | **0.72 ± 0.20d** | **0.53 ± 0.21d** | **1.70 ± 0.11bc** | **1.09 ± 0.36d** | **<LOD** | **2.02 ± 0.27ab** | **1.15 ± 0.19cd** | **1.16 ± 0.22cd** | **2.34 ± 0.21a** | **0.88 ± 0.26d** | **0.82 ± 0.15d** | **1.71 ± 0.32b** |
| Kaempferol | **0.14 ± 0.04b** | **n.d.** | **1.83 ± 0.28a** | **n.d.** | **n.d.** | **2.43 ± 0.50a** | **n.d.** | **n.d.** | **n.d.** | **n.d.** | **n.d.** | **2.12 ± 0.67a** | **n.d.** | **n.d.** | **n.d.** |
| Myricetin | **n.d.** | **n.d.** | **n.d.** | **n.d.** | **n.d.** | **n.d.** | **n.d.** | **n.d.** | **n.d.** | **0.34 ± 0.10b** | **n.d.** | **1.41 ± 0.27a** | **0.50 ± 0.09b** | **0.22 ± 0.05b** | **1.69 ± 0.23a** |
| Quercetin | **n.d.** | **n.d.** | **0.48 ± 0.06c** | **0.24 ± 0.13c** | **n.d.** | **2.00 ± 0.20a** | **n.d.** | **n.d.** | **n.d.** | **0.41 ± 0.35c** | **n.d.** | **1.72 ± 0.29a** | **0.29 ± 0.07c** | **n.d.** | **1.24 ± 0.19b** |
| Rutin | **n.d.** | **n.d.** | **n.d.** | **n.d.** | **n.d.** | **n.d.** | **n.d.** | **n.d.** | **n.d.** | **n.d.** | **n.d.** | **n.d.** | **LL** | **0.93 ± 0.06b** | **LL** |
| TF | **1.91 ± 0.34c** | **1.75 ± 0.33c** | **2.47 ± 0.78c** | **5.24 ± 0.73b** | **1.91 ± 0.66c** | **19.56 ± 0.80a** | **n.d.** | **n.d.** | **n.d.** | **n.d.** | **n.d.** | **n.d.** | **n.d.** | **n.d.** | **n.d.** |
| TF3G | **8.00 ± 0.52c** | **7.65 ± 0.46c** | **12.65 ± 0.95b** | **11.88 ± 0.43b** | **5.66 ± 0.50d** | **43.35 ± 0.34a** | **n.d.** | **n.d.** | **n.d.** | **n.d.** | **n.d.** | **n.d.** | **n.d.** | **n.d.** | **n.d.** |
| TF3′G | **0.96 ± 0.44b** | **0.66 ± 0.83b** | **1.86 ± 1.10b** | **1.77 ± 1.08b** | **0.77 ± 0.55b** | **10.92 ± 1.07a** | **n.d.** | **n.d.** | **n.d.** | **n.d.** | **n.d.** | **n.d.** | **n.d.** | **n.d.** | **n.d.** |
| TF3,3′DG | **5.73 ± 0.23c** | **5.85 ± 0.14c** | **9.44 ± 0.35b** | **5.73 ± 0.13c** | **3.23 ± 0.06d** | **20.33 ± 0.29a** | **n.d.** | **n.d.** | **n.d.** | **n.d.** | **n.d.** | **n.d.** | **n.d.** | **n.d.** | **n.d.** |

^a^ BTE, black tea extract; CTE, co-oxidized tea extract; GTE, green tea extract; MTE, microbial fermented green tea extract; PTE, post-fermented green tea extract; ^b^ Refer to the main article for the abbreviations of compounds, and the unit is presented as mg compound/g dry weight of extracts (DW_EX_). ^c^ T, total extract; Fw, hydrophilic fraction; and Fo, hydrophobic fraction. ^d^ n.d.: not detected. ^e^ <LOD: low limit of quantification. Concentrations are expressed as mg/g dry weight sample, and results are shown as means ± standard error of the mean (n > 3). Different lowercase letters in the same row indicate significant differences (*p* < 0.05) of the means.

**Table** **S2**. Flavonol and flavone glycosides relative content^a^ of the five tea extracts

^a^ Data expressed as % mean peak area based on the mean peak area of GTE peak in the same row (n = 3).
* Refer to the peaks of main article **Table 1**.

**Table** **S3**. Total flavonoid and phenolic contents, and antioxidant capacities of the five tea extracts and their fractions

| Extract and fraction | TFC^a^ | TPC^b^ | ABTS^c^ | DPPH^c^ | FRAP^c^ |
| --- | --- | --- | --- | --- | --- |
| BTE-Fo | 81.3 ± 3.8f | 167.5 ± 2.5g | 282.6 ± 17.0i | 207.2 ± 13.4f | 110.1 ± 6.9g |
| BTE-Fw | 82.7 ± 5.1ef | 303.3 ± 1.4cd | 599.9 ± 23.8efg | 365.2 ± 19.1d | 239.5 ± 5.5d |
| BTE-T | 78.8 ± 2.3f | 275.0 ± 9.0de | 524.6 ± 18.6fgh | 296.5 ± 29.5de | 214.0 ± 8.0de |
| CTE-Fo | 115.6 ± 3.6ab | 280.8 ± 3.8cde | 490.0 ± 9.8gh | 313.1 ± 6.2de | 172.3 ± 8.3ef |
| CTE-Fw | 79.5 ± 3.3f | 314.2 ± 18.8c | 651.7 ± 23.8def | 313.8 ± 66.1de | 246.3 ± 3.3d |
| CTE-T | 100.0 ± 1.1cd | 311.7 ± 9.5cd | 636.9 ± 21.1def | 330.4 ± 38.2de | 233.7 ± 8.5d |
| GTE-Fo | 121.4 ± 2.3a | 232.5 ± 6.6f | 432.0 ± 4.3h | 279.8 ± 18.0def | 234.8 ± 7.6d |
| GTE-Fw | 105.6 ± 8.3bcd | 465.8 ± 20.8a | 1160.4 ± 38.7a | 880.4 ± 14.4a | 622.2 ± 8.3a |
| GTE-T | 113.2 ± 7.6abc | 438.3 ± 21.0a | 1071.5 ± 23.1ab | 809.6 ± 7.2a | 525.6 ± 38.6b |
| MTE-Fo | 95.2 ± 4.1de | 258.3 ± 8.0ef | 418.4 ± 15.0h | 259.0 ± 7.2ef | 237.7 ± 5.0d |
| MTE-Fw | 103.8 ± 3.6bcd | 438.3 ± 13.8a | 904.8 ± 52.2c | 659.6 ± 19.1b | 537.7 ± 5.0b |
| MTE-T | 115.0 ± 1.1ab | 355.8 ± 21.6b | 764.1 ± 9.8d | 551.3 ± 54.5c | 440.3 ± 30.2c |
| PTE-Fo | 47.2 ± 5.2g | 150.0 ± 4.3g | 234.4 ± 13.4i | 196.0 ± 10.1f | 131.9 ± 21.6fg |
| PTE-Fw | 109.4 ± 5.2abc | 391.7 ± 14.2b | 964.1 ± 13.4bc | 705.4 ± 28.9b | 533.2 ± 9.6b |
| PTE-T | 108.4 ± 3.1abcd | 358.3 ± 6.3b | 682.6 ± 142.3de | 484.6 ± 19.1c | 428.5 ± 33.6c |

Different lowercase letters in the same column indicate significant differences (p < 0.05) of the means. ^a^ TFC, total flavonoid content (unit: mg catechin equivalents/g DW_EX_), ^b^ TPC, total flavonoid content (unit: mg gallic acid equivalents/g DW_EX_). ^c^ ABTS, 2,2-azino-bis(3-ethylbenzothiazoline-6-sulfonic acid) radical scavenging assay; DPPH, 1,1-diphenyl-2-picrylhydrazyl radical scavenging assay; FRAP, ferric reducing antioxidant power assay (unit: mg vitamin C equivalents/g DW_EX_).

**Table** **S4**. Correlation result of multivariate analysis among total flavonoid and phenolic contents, antioxidant capacities, and phenolic compounds in five tea extracts and their fractions

Green numbers indicate statistically significant (*p* <0.05). Red bar and blue bar indicate negative and positive values, respectively. Refer to the main article for the abbreviations of chemical compounds.

**Table** **S5**. Correlation result of multivariate analysis for the anticancer effects and antioxidant capacity and phenolic compounds of three tea extracts and their fractions (GTE, CTE, and BTE)

Green numbers indicate statistically significant (*p* <0.05). NA means not available.
DLD-1, colorectal adenocarcinoma cell line; E0771, murine breast cancer cell line.


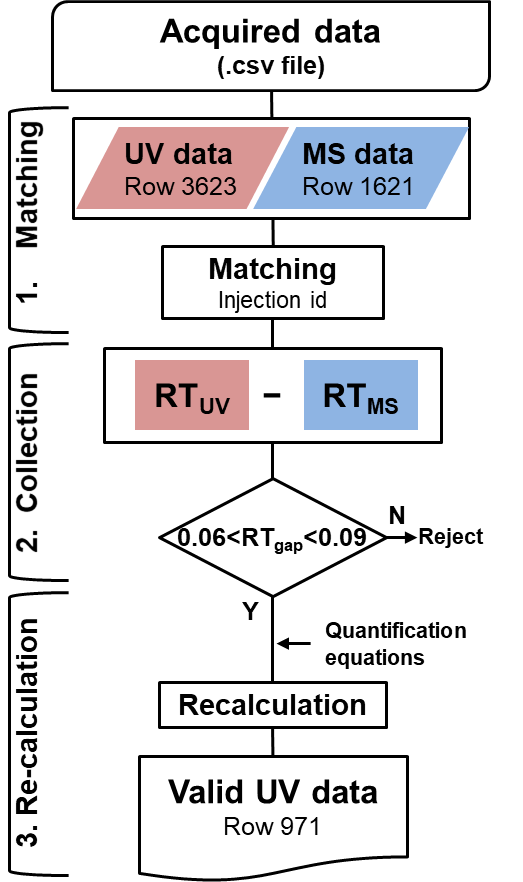


**Figure** **S3**. Process flow for collection of valid UV data


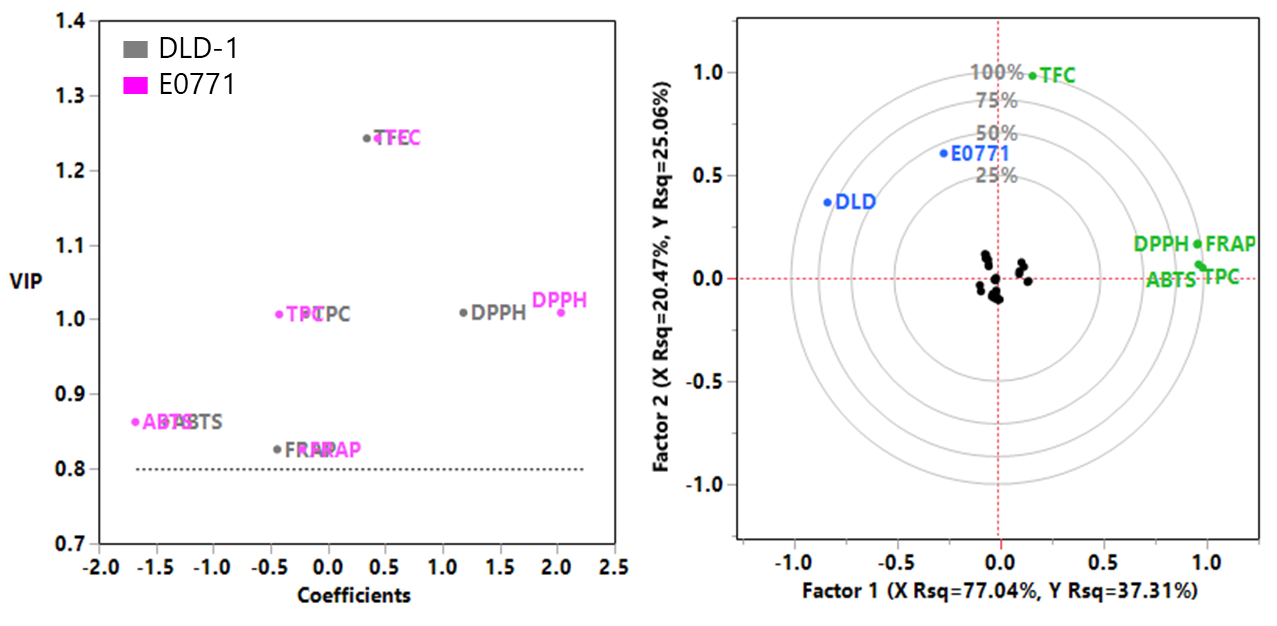


**Figure** **S4**. Correlation loading plot of PLS-DA for anticancer effect by antioxidant capacities, and total flavonoid and phenolic contents. VIP, variable importance in projection, The Factor 1 and factor 2 are constructed to account for the correlation or covariance between the observed variables. Factor rotation was used to change the reference axes of the factors to increase their interpretability.

**Script** **1**. Coded script for collecting valid UV data based on MS data

| *## Install packages*  install.packages("dplyr")  install.packages("ggplot2")  install.packages("fuzzyjoin")  *## Import Library*  library("dplyr")  library("ggplot2")  library("fuzzyjoin")  *## Read mass data and select column*  df_MS1 <- read.csv("Tea_MS1.csv")  df_MS1 <- df_MS1 %>%  select(SMP, Injection_Id, Peak_Name, Retention_Time) %>%  mutate(RT_min = Retention_Time+0.06, RT_max=Retention_Time+0.09) %>%  select(SMP, Injection_Id, Peak_Name, RT_min, RT_max)  df_MS2 <- read.csv("Tea_MS2.csv")  df_MS2 <- df_MS2 %>%  select(SMP, Injection_Id, Peak_Name, Retention_Time) %>%  mutate(RT_min = Retention_Time+0.06, RT_max=Retention_Time+0.09) %>%  select(SMP, Injection_Id, Peak_Name, RT_min, RT_max)  *## Read UV data and select column*  df_UV1 <- read.csv("Tea_UV_275.csv")  df_UV1 <- df_UV1 %>%  select(Inj_Id, Retention_Time, Area, Dilution)  df_UV2 <- read.csv("Tea_UV_365.csv")  df_UV2 <- df_UV2 %>%  select(Inj_Id, Retention_Time, Area, Dilution)  *## Join data frame with conditions*  df_MS3 <- fuzzy_inner_join(df_MS1, df_UV1,  by=c("Injection_Id"="Inj_Id","RT_min"="Retention_Time", "RT_max"="Retention_Time"),  match_fun=list(`==`, `<=`, `>=`)) %>%  select(SMP, Injection_Id, Peak_Name, Retention_Time, Area, Dilution)  df_MS4 <- fuzzy_inner_join(df_MS2, df_UV2,  by=c("Injection_Id"="Inj_Id","RT_min"="Retention_Time", "RT_max"="Retention_Time"),  match_fun=list(`==`, `<=`, `>=`)) %>%  select(SMP, Injection_Id, Peak_Name, Retention_Time, Area, Dilution)  *## Re-calculation of compounds concentration*  df_MS3 <- df_MS3 %>%  mutate(Re_amount = ifelse(Peak_Name =="C",(Area - 6.88e+002)/(5.83e+003),  ifelse(Peak_Name =="Caffeine",(Area - 7.52e+002)/(2.37e+004),  ifelse(Peak_Name =="CG",(Area - 6.15e+002)/(1.22e+004),  ifelse(Peak_Name =="EC",(Area - 4.99e+002)/(4.41e+003),  ifelse(Peak_Name =="ECG",(Area - 1.03e+003)/(1.28e+004),  ifelse(Peak_Name =="EGC",(Area - 7.11e+002)/(3.18e+003),  ifelse(Peak_Name =="EGCG",(Area - 5.88e+002)/(1.04e+004),  ifelse(Peak_Name =="Gallic acid",(Area - 4.80e+002)/(1.26e+004),  ifelse(Peak_Name =="GC",(Area - 1.02e+002)/(1.81e+003),  ifelse(Peak_Name =="GCG",(Area - 7.18e+002)/(9.73e+003),  ifelse(Peak_Name =="TF1",(Area - 1.07e+003)/(1.15e+004),  ifelse(Peak_Name =="TF2a",(Area - 4.47e+002)/(8.37e+003),  ifelse(Peak_Name =="TF2b",(Area - 2.40e+003)/(1.47e+004),  ifelse(Peak_Name =="TF3",(Area - 2.02e+002)/(1.62e+004),NA)))))))))))))))  df_MS3 <- df_MS3 %>%  mutate(Re_value = Re_amount*1000/Dilution)  df_MS4 <- df_MS4 %>%  mutate(Re_amount = ifelse(Peak_Name =="Apigenin",(Area - 7.60e+002)/(1.77e+004),  ifelse(Peak_Name =="Isoquercitrin",(Area - 6.30e+002)/(1.32e+004),  ifelse(Peak_Name =="Kaempferol",(Area - 7.14e+002)/(2.32e+004),  ifelse(Peak_Name =="Myricetin",(Area - 2.60e+002)/(3.06e+004),  ifelse(Peak_Name =="Quercetin",(Area - 3.85e+002)/(2.59e+004),  ifelse(Peak_Name =="Rutin",(Area - 5.07e+002)/(1.05e+004),NA)))))))  df_MS4 <- df_MS4 %>%  mutate(Re_value = Re_amount*1000/Dilution)  *## Combine data frames*  df_Result <- bind_rows(df_MS3, df_MS4)  write.csv(df_Result, file = "5Tea_results.csv") |
| --- |

Reference

1. Benzie, I.F.; Strain, J.J. The ferric reducing ability of plasma (FRAP) as a measure of “antioxidant power”: the FRAP assay. *Anal. Biochem.* **1996**, *239*, 70-76.
